# Getting It Right in Restrictive Lung Disease

**DOI:** 10.3390/jcm12103353

**Published:** 2023-05-09

**Authors:** Annalisa Carlucci, Barbara Fusar Poli

**Affiliations:** 1 Dipartimento di Medicina e Chirurgia, Università Insubria, 21100 Varese, Italy; 2 Pneumologia Riabilitativa Istituti Clinici Scientifici Maugeri, 27100 Pavia, Italy

**Keywords:** chest wall disease, scoliosis, neuromuscular disease, ALS, MND, hypoventilation, non-invasive ventilation, mouthpiece ventilation

## Abstract

Restrictive lung disease (predominantly in patients with neuromuscular disease (NMD) and ribcage deformity) may induce chronic hypercapnic respiratory failure, which represents an absolute indication to start home NIV (HNIV). However, in the early phases of NMD, patients may present only diurnal symptoms or orthopnoea and sleep disturbances with normal diurnal gas exchange. The evaluation of respiratory function decline may predict the presence of sleep disturbances (SD) and nocturnal hypoventilation that can be respectively diagnosed with polygraphy and PCO_2_ transcutaneous monitoring. If nocturnal hypoventilation and/or apnoea/hypopnea syndrome are detected, HNIV should be introduced. Once HNIV has been started, adequate follow-up is mandatory. The ventilator’s built-in software provides important information about patient adherence and eventual leaks to correct. Detailed data about pressure and flow curves may suggest the presence of upper airway obstruction (UAO) during NIV that may occur with or without decrease in respiratory drive. Etiology and treatment of these two different forms of UAO are different. For this reason, in some circumstances, it might be useful to perform a polygraph. PtCO_2_ monitoring, together with pulse-oximetry, seem to be very important tools to optimize HNIV. The role of HNIV in neuromuscular disease is to correct diurnal and nocturnal hypoventilation with the consequence of improving quality of life, symptoms, and survival.

## 1. Introduction

Hypercapnic chronic respiratory failure in respiratory disease usually represents a reason to consider prescription of home non-invasive ventilation (HNIV). When to start it and how to get it right depends on the underlying chronic respiratory disease and the available clinical evidence. The aim of this review is to focus on indications for and methods of using HNIV in patients with chronic respiratory failure due to restrictive lung disease (predominantly patients with neuromuscular disease and ribcage deformity). We will start with a clinical case to follow a possible “journey” of a patient with chronic respiratory failure who is a possible candidate for HNIV.

### A Typical Patient’s Clinical Journey

Our case is a 53-year-old patient who was sent for respiratory consultation after having received the diagnosis of amyotrophic lateral sclerosis (ALS). He presented with four to five months of walking problems, proximal arm weakness, and recently reduced physical activity, while still maintaining a sedentary job. When questioned, he referred to shortness of breath during activities of daily living (washing himself, eating), difficulty sleeping and frequent awakenings during the night, morning headaches, and a sense of confusion. The patient dismissed these symptoms and contributed them to the anxiety induced by his recent diagnosis. The respiratory history was unremarkable, the respiratory assessment was normal, and oxygen saturation (SpO_2_) was 95% on room air. Arterial blood gases (ABGs) showed a PaO_2_ of 75 mmHg, a PaCO_2_ of 37 mmHg, pH 7.45 (while breathing room air), and HCO_3_^−^ 28 mmol/L. Respiratory function tests showed a vital capacity (VC) of 70% of the predicted value, with normal ratio of forced expiratory volume in the first second to VC. Spirometry repeated in a supine position showed a VC drop of 33%. Polysomnography was performed, showing mild obstructive sleep apnea (OSA) with an apnea hypopnea index (AHI) of 12 events per min. Mean SpO_2_ was 93%, with a nadir of 89% and 5% of sleep time spent with SpO_2_ < 90%. Transcutaneous monitoring of PCO_2_ (PtCO_2_) was performed by placing an ear sensor: PtCO_2_ baseline was 42 mmHg, and PtCO_2_ median value was 52 mmHg, with a maximum of 57 mmHg and 120 min of sleep time spent with a PtCO_2_ > 50 mmHg. The next step is identifying where we go from here.

## 2. Methods: Literature Review

We conducted a comprehensive PubMed search of full-length articles in English using the following key terms: “chronic hypercapnic respiratory failure” or “restrictive disease” or “neuromuscular disease” or amyotrophic lateral sclerosis” and “home non-invasive ventilation.” We excluded articles published in non-peer-reviewed journals. Papers were independently reviewed by the two authors, and those judged more informative for the purpose of the review were retained.

## 3. Results

### 3.1. When Is the Right Time to Start Home NIV?

**Figure 1:**
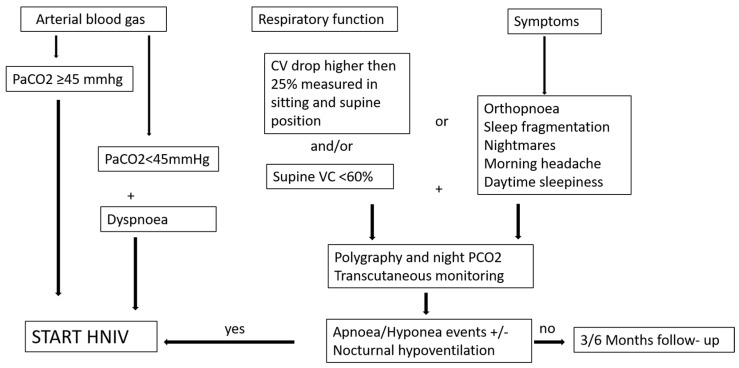
Shows possible management of patients affected by Neuromuscular disease. Arterial blood gas (in particular, PaCO_2_ levels), combined with respiratory function and symptoms, may suggest the right time to introduce HNIV. If the patient presents with normocapnia, with compromised vital capacity (VC) or nocturnal symptoms, a polygraphy and PCO_2_ trancutaneous monitoring should be performed to identify a nocturnal hypoventilation and other important criteria to start HNIV.

Restrictive diseases (RDs) are characterized by restrictive respiratory dysfunction due to respiratory muscle weakness from neuromuscular diseases (NMDs) and/or chest wall deformity. The natural history of RDs leads first to sleep disturbances (SDs) (apnea/hypopnea and/or hypoventilation syndrome) and finally leads to daytime hypoventilation [1]. The leading causes of death and major morbidity are respiratory infections and respiratory failure [2]. Home non-invasive ventilations (HNIVs) significantly improve survival in ALS and Duchenne muscular dystrophy (DMD), while improvement in quality of life, diurnal symptoms, SD, and quality of sleep was shown in all the other pathologies under the umbrella of NMDs [3]. In chest wall diseases, there are no published randomized controlled trials comparing HNIV to oxygen therapy. However, in this subgroup of patients, a prospective controlled non-randomized study, showed better survival with HNIV compared to oxygen alone, even when adjusting for age, gender, blood gas levels, and associated respiratory diseases [4]. For these reasons, HNIV is suggested by many scientific societies for treatment of chronic hypercapnic respiratory failure in RD. However, there is weak evidence about the right timing for starting HNIV, and this has led to a lack of definitive consensus. Development of chronic hypercapnic respiratory failure defined as diurnal hypercapnia at arterial blood gases (ABGs) (pCO_2_ > 45 mmHg) surely represents an indication to start HNIV in RD. However, patients who still maintain normal diurnal arterial pCO_2_ may also need intervention. A cut-off of VC < 50% of the predicted value has been suggested as the only criteria to start HNIV, or a VC < 80% associated with symptoms [5]. However, many years ago, a study enrolling DMD patients in the early stage of functional decline who had not yet developed daytime respiratory failure showed a worse outcome with a high mortality rate when compared with non-ventilated patients [6]. This study had a lack of data about adherence to therapy and a lack of follow-up clinical data. Therefore, it was suggested that low adherence even when hypoventilation developed might explain the higher mortality. This assumption is supported by the more recent finding in ALS patients with a very low adherence to HNIV (lower than 3 h/day) who had a higher mortality when it was started earlier [7]. On the other hand, some years ago, Ward et al. showed that in a heterogeneous group of RD without diurnal hypercapnia, HNIV can delay development of diurnal hypercapnia when started in patients with nocturnal hypoventilation alone as detected by PtCO_2_ monitoring [8]. Hypoventilation was defined in this study as detection of a peak PtCO_2_ of > 49 mmHg. Based on this study, nocturnal hypoventilation may be considered as criteria to start HNIV. However, transcutaneous monitoring of PCO_2_ is expensive, and that limits its use to systematically screening NMD patients for nocturnal hypoventilation. We need predictors of SD and hypoventilation. Symptoms such as dyspnea, orthopnea, morning headaches, sleep fragmentation, and excessive daytime sleepiness are warning signs of SD and should be investigated. It has also been shown that there is a good correlation between the respiratory function decline, in particular vital capacity (VC), measured in supine position and SD [9]. In particular, a supine VC < 60% seems to highly correlate with SD. Also, the detection of a drop greater than 25% between VC measured in sitting and supine positions has been shown to correlate with diaphragmatic dysfunction and might suggest the presence of nocturnal hypoventilation [10]. We can, therefore, suggest using symptoms and spirometry to detect the right time to screen patients for SD with polygraphy and transcutaneous CO_2_ monitoring. A proposition of a decision tree to start HNIV in these patients is illustrated in Figure 1.

Diurnal and/or nocturnal hypoventilation and or apnea/hypopnea syndrome should be detected to start HNIV, as already suggested for ALS patients [11]. Another crucial point is the definition of nocturnal hypoventilation: prevalence of nocturnal hypoventilation, in fact, may vary widely according to definitions provided. A nocturnal pulse-oximetry showing oxygen saturation (SpO_2_) ≤ 88% for 5 consecutive minutes was first proposed [12]. However, SpO_2_ is not useful when the patient has lung disease or is using supplemental oxygen therapy. Moreover, in patients with restrictive diseases, capnography was shown to be more sensitive in detecting nocturnal hypoventilation, since about 30% of patients with normal nocturnal SpO_2_ had manifested nocturnal hypercapnia [13]. The American Academy of Sleep Medicine proposed the following criteria to define hypoventilation: PtCO_2_ ≥ 55 mmHg for more than 10 min or an increase in PtCO_2_ ≥ 10 mmHg compared to an awake supine value, to a value exceeding 50 mmHg for more than 10 min [14]. However, in ALS patients, a marked difference in the prevalence of hypoventilation has been described due to the different definitions proposed in the literature. Moreover, the most commonly used definitions of hypoventilation lead to variable ability in predicting clinically relevant outcomes, that is, the need to initiate HNIV [15]. In particular, a nocturnal peak of PtCO_2_ higher than 49 mmHg seems to be the best predictor of needing HNIV [15]. Metabolic alkalosis (with bicarbonates > 27 mEq/L) on ABGs may also suggest the development of nocturnal hypoventilation after having excluded possible confounding factors such as associated metabolic diseases (i.e., diabetes) and drugs (i.e., diuretics).

A possible grey zone In defining when to start HNIV is represented by patients who complain of symptoms such as dyspnea but do not show diurnal or nocturnal hypoventilation. Many years ago, a physiological study [16] showed that dyspnea in RD may be present even in the absence of significant functional impairment (mean VC 75%) and diurnal hypercapnia. Dyspnea significantly correlates with a reduced compliance of the respiratory system and is responsible for neuro-ventilatory uncoupling. In fact, lung and chest wall compliance have been shown to worsen in NMD, resulting in increasing elastic load on respiratory muscles and lower lung volumes. Indeed, the decrease in vital capacity, associated with the progressive loss of inspiratory and expiratory muscle function, leads to progressive atelectasis and alteration of tissue mechanics, finally determining lung stiffness [16]. The introduction of HNIV, in this case, may improve respiratory muscles’ efficiency by supporting them and reducing elastic load by increasing tidal volume and alveolar recruitment.

### 3.2. Back to Our Patient Case and Their Journey

**Figure 2:**
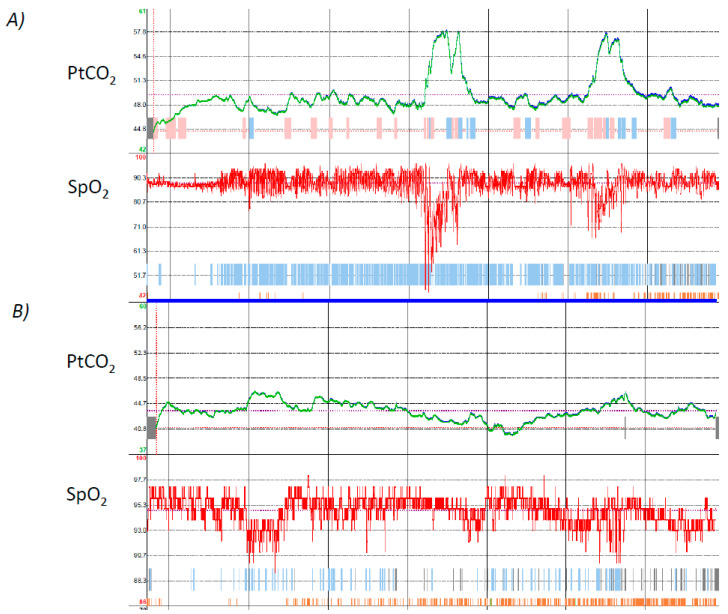
Shows transcutaneous monitoring of a patient with ALS during spontaneous breathing (**A**) and during HNIV (**B**). In (**A**) note a progressive increase of PtCO_2_ at the start of recording time and two further abrupt increases that may represent the REM stage of sleep. SpO_2_ trace shows recurrent oxygen desaturation events (likely apnea/Hypopnea events) during the night with two longer and more profound oxygen desaturations (likely REM-related alveolar hypoventilation). During HNIV (**B**) the PtCO_2_ is stable around a normal value and SpO_2_ has significantly improved.

The patient started nocturnal NIV with spontaneous/timed mode with the following parameters: inspiratory positive airway pressure (IPAP) = 16 cmH_2_O, expiratory positive airway pressure (EPAP) = 4 cmH_2_O, back-up rate = 12 breaths per minute (BPM), and an oro-nasal mask. We monitored pressure and flow waveforms from the built-in software of the ventilator combined with plugged-in pulse-oximetry. The download showed several episodes of oxygen desaturation. Detailed data showed that these episodes were preceded by intermittent flow reduction as already described in the presence of upper airway obstruction (UAO) [17]. Based on these results, the EPAP was increased to 12 cmH_2_O. However, there was not a significant reduction in the number of events. We therefore performed a polygraphy that confirmed UAO in the absence of ventilatory drive. In addition, PtCO_2_ monitoring showed a mean PtCO_2_ lower than 37 mmHg, with a nadir of 28 mmHg. The IPAP was reduced along with the EPAP, allowing a significant reduction of respiratory events during NIV. A picture of transcutaneous monitoring before starting HNIV and at discharge of the patient is reported in Figure 2.

A three-month evaluation showed the patient to be in a stable clinical condition with normal ABG with regular 8-h use of HNIV (downloaded with device’s SD card); no leaks were detectable during most of the night, and the sleep quality was preserved. However, after 6 months, the patient presented with neurological worsening, consisting of a reduction in peripherical strength, weight loss, dyspnea, and evidence of diurnal hypercapnia on ABGs (PaO_2_ = 70 mmHg, PaCO_2_ = 48 mmHg, pH = 7.43, HCO_3_^−^ 30.5 mmol/L), which were taken while the patient was spontaneously breathing on room air. Ventilator built-in software also showed a significantly increased daily ventilator use to 14 to 16 h/day. Nocturnal mean PtCO_2_ was 45 mmHg, and time spent with pCO_2_ > 49 mmHg was five percent during NIV. These data suggested the inability of the patient to sustain spontaneous breathing for a long time. We needed to increase the hours of ventilation during the daytime, possibly by considering another interface. In this instance, we successfully initiated mouth-piece ventilation (MPV). The patient’s ventilator was set with a double profile. This allowed us to set a nocturnal profile with the oro-nasal mask and a daytime profile with MPV mode.

### 3.3. How to Ventilate: Settings and Objective Measures of Successful Ventilation

A recent European survey [18] showed that in RD, the most used mode of ventilation is the pressure-controlled mode (PCV). In patients with a residual respiratory drive, this mode is more physiologically similar to their spontaneous ventilation than the volume-controlled mode (VCV), ensuring a variability of volume according to different efforts of the patient. Moreover, nonintentional leaks are better compensated for compared to VCV [19]. On the other hand, VCV allows the patient to perform air-stacking to assist airway clearance and is able to overcome changes in respiratory mechanics, above all the increase of inspiratory resistance. Volume-target pressure-controlled mode (VTPCV) is a hybrid ventilation mode that aims to combine the advantages of the two conventional ventilatory modes: maintaining a more comfortable mode by guaranteeing a pre-set volume irrespective of changes in respiratory system elastance (i.e., body position or pulmonary mechanics). In ALS patients, a comparison between PCV and VCV did not show a significant difference in terms of survival [20]. However, none of the few randomized trials in neuromuscular diseases have compared long-term VTPCV modes with conventional PCV. Moreover, short-term studies evaluating sleep quality and nocturnal PCO_2_ led to different and contrasting results [19]. We should also be aware of pitfalls and complications associated with VTPCV. In fact, it has been shown that the VTPCV mode used with a single limb circuit with valve was responsible for under-assistance in the presence of leaks [21].

The aims of HNIV are to improve symptoms and to reverse respiratory failure, which means normalizing daytime ABGs and improving nocturnal hypoventilation. However, concerns are still raised about the definition of “improving” nocturnal hypoventilation. Some years ago, Gonzales-Bermejo showed that in a group of ALS patients starting HNIV, one month after starting HNIV, almost 50% of the patients were poorly ventilated, as defined by the presence of more than 5% of the sleep time spent with SpO_2_ below 90% with nocturnal pulse-oximetry [12]. Poor ventilation, in this study, significantly correlated with a worse survival rate. However, pulse-oximetry was not sensitive enough in detecting nocturnal hypoventilation, while transcutaneous PCO_2_ monitoring was shown to have a higher sensitivity to detect this [22]. Moreover, in a retrospective study, the authors found that residual hypoventilation assessed by capnometry is significantly associated with negative outcomes in adult ventilated NMD patients, while oximetry is not [23]. In this latter study, the definition of residual nocturnal hypercapnia as PtCO_2_ > 49 mmHg during ≥10% of the total recording time was found to be the more sensitive criterion to identify nocturnal hypoventilation. The authors found that 27% of enrolled patients presented this criterion of hypoventilation despite normal diurnal ABGs, and this was associated with the worst outcomes.

### 3.4. How to Set a Ventilator

**Figure 3:**
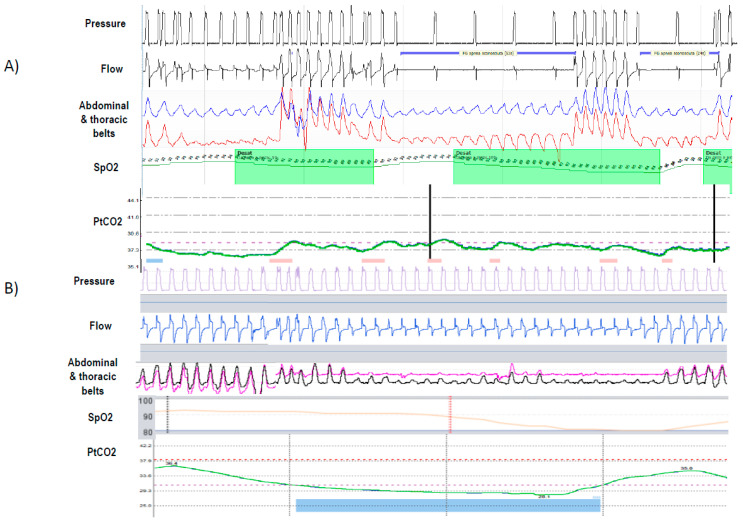
Shows upper airway obstruction (UAO). (**A**) Without reduction of ventilatory drive: (1) sudden reduction in flow amplitude; (2) phase opposition in thoracic and abdominal belts; (3) decrease in SpO_2_ 15 sec after the UAO; (**B**) with decrease in ventilatory command: (1) progressive decrease in flow; (2) significant reduction/disappearance of thoracoabdominal movements and change to back-up ventilation indicating upper airway closure with reduced ventilatory command; (3) a significant reduction of PtCO_2_ preceded the event.

It is a common perception that PaCO_2_ reduction in patients with NMD is easy to achieve and that these patients do not need high pressure support (PS) values to improve alveolar ventilation. However, in a recent post-hoc analysis of a previous randomized controlled study, Leotard and co-workers showed that high tidal volumes (VT), were more significantly associated with a lower mean nocturnal PtcCO_2_ than higher PS alone [24], and there was not a correlation between VT and PS. This means that in patients with NMD, similar PS levels might result in highly variable changes in alveolar ventilation depending on the heterogeneity of ventilatory mechanics. Monitoring PtCO_2_ may be the right way to better titrate settings. Another possible reason for the poor correlation between PS and VT is the presence of leaks and UAO. It has been demonstrated that these two events account for almost 80% of the causes of persistent desaturation during HNIV in NMD [12], and they also represent the most frequent etiology of the most common patient-ventilator asynchronies (PVAs) during nocturnal NIV [25]. For this reason, skill in the use of built-in software should be acquired to monitor HNIV in RD patients [26]. The built-in software allows you to monitor in detail items such as adherence, leaks, tidal volume, and residual respiratory events, as well as conduct analysis of detailed data for flow and pressure waveforms. Moreover, some ventilators can be connected to oximetry, transcutaneous monitoring, and sometimes thoracic and abdominal effort belts, giving information similar to what a polygraph gives. When residual nocturnal UAO is present during HNIV in RD, several different etiologies should be considered [27]. In fact, apnea/hypopnea events during NIV, above all in RD, may occur with or without decrease in respiratory drive [17]. These two different phenomena are correlated with different etiology and consequently need different treatments [17]. In particular, UAO without respiratory drive may be associated with an intrinsic vocal cord dysfunction or with a mechanism of glottis closure due to excessive respiratory support leading to hyperventilation and hypocapnia. In this case, the setting of the ventilator should be modified in favor of lower instantaneous inspiratory flow by decreasing the pressurization rate (time to reach the set level of PS) and the PS level. It is not necessary to increase the EPAP level, which, on the contrary, might be decreased if no other type of obstructive events should be corrected. Conversely, obstruction with respiratory drive may be due to the classic oropharyngeal collapse. In this case, an increase of EPAP should be the first attempt in the setting review. When all of these corrective measures fail, a more detailed analysis of the cause of obstruction by videolaryngoscopy during NIV has been proposed [27]. Another typical case of obstruction with active drive, in fact, may be induced by the oro-nasal mask by pushing the soft palate and uvula backward. In this case, neither the increase of EPAP nor that of the PS level is able to improve the inspiratory flow, but a switch to a nasal mask is suggested when possible. A positional therapy that forces the patient to maintain a lateral position during the night may reduce and sometimes completely resolve the events and could be an alternative. Because UAO with and without drive requires different treatment, sometimes belts are needed to discriminate the two different etiologies, as shown in Figure 3.

There are no insights into the utility of back-up rate. One recent study compared a spontaneous bilevel mode with a spontaneous/timed one in ALS patients [28]. They showed a superiority of back-up frequency in improving gas exchanges at the same IPAP and EPAP level. However, no differences in the two modes have been shown on average in terms of sleep architecture and nocturnal asynchronies. Nevertheless, authors highlighted the importance of well titrating and monitoring detailed data from ventilators, because sometimes an apparent well-controlled ventilation (majority of time spent on back-up rate) can mask asynchronies, above all ineffective efforts. Likewise, no data about the possible impact of different pressurization rates are available. A proposal for the setting of HNIV considering the objective of ventilation is reported in Table 1.

### 3.5. Mouth-Piece Ventilation (MPV)

**Figure 4:**
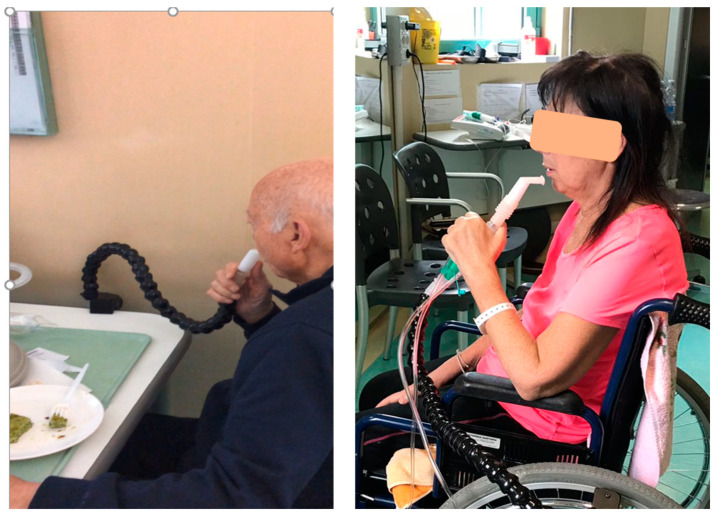
Two photographs of patients using mouthpiece ventilation (MPV). The left-hand picture shows an open MPV circuit; the patient breathes in via the circuit and then disengages from the mouthpiece to breathe out. The picture on the right shows an MPV circuit with an exhalation valve; in this situation, the patient can breathe in and out via the circuit. The exhaled air will be via the exhalation valve.

When daytime hypercapnia persists despite nocturnal normocapnia, or diurnal dyspnea or breathlessness is present while eating or when walking, diurnal ventilation, possibly using another interface to avoid pressure sores, should be considered. MPV (Figure 4) may be an interesting alternative to nasal and oronasal mask ventilation. MPV is an open ventilation system that can be used on demand (in patients with preserved ventilator autonomy) or as controlled ventilation (when the ability to generate an effort or the respiratory drive is almost completely abolished) [29]. MPV can be used in PCV or VCV. VCV is preferred when MPV is used also to perform air staking to improve cough efficiency. Because of the possible presence of major leaks and the fact that the patient may use it only when he needs to, with long-lasting pauses between breaths, managing alarms is a challenge with MPV [30]. For this reason, most life-support home ventilators are equipped with a dedicated mode generally called “MPV mode.” By creating different profiles on the ventilator, a different setting for different interfaces used or nocturnal and diurnal ventilation may be considered.

### 3.6. Beyond HNIV

People with neuromuscular diseases may have difficulty coughing and clearing mucous from the airways, placing them at risk of choking, recurrent chest infections, and acute respiratory failure episodes. Early and appropriate use of cough augmentation techniques, such as mechanical cough assist (a device that clears secretions by applying a positive pressure to the airway then rapidly shifting to a negative pressure) combined with HNIV can improve respiratory function, ensuring fewer complications, reducing emergency department visits, and decreasing acute care (including intensive care) admissions [31]. It has been suggested that adults with NMD require a peak expiratory cough flow of more than 270 L/min when well to account for the expected decline in cough flows during intercurrent respiratory infections. Cough augmentation with mechanical cough assist should, therefore, be indicated if PCF falls below 270 L/min [29]. However, despite the strong physiologic backgrounds supporting the association of the mechanical in-exsufflation with HNIV in neuromuscular patients with a weak cough, more evidence is needed to identify the real effectiveness of it in addressing respiratory morbidity and resultant health care utilization and costs for individuals with neuromuscular disorders [32].

## 4. Summary

In RD, HNIV improves quality of life, symptoms, and survival. Hypoventilation, both diurnal and nocturnal, represents the most important reason to start HNIV, together with symptoms and respiratory function impairment. Transcutaneous monitoring of PCO_2_ and accompanying pulse-oximetry represent the most sensitive tools to detect hypoventilation, and their use is also suggested to monitor the effects of HNIV. Particular skill is required in the use of built-in software to monitor HNIV, in particular in the interpretation of flow and pressure curves to detect leaks and UAO, which represent the most important causes of inefficient ventilation. Polygraphy under HNIV may be required to better understand the reason for UAO and treat it.

## Figures and Tables

**Table 1:** Proposal for setting of HNIV in NMD.

Ventilator Parameter	Setting	Objective	Possible Problems
**Pressure Support** **(cmH_2_O)**	Starting from 8 (normally no need for high PS level)	Improve diurnal pCO_2_ and nocturnal PtCO_2_; treat hypopnea	Too high value may induce glottic closure and/or leaks
**Pressurization Rate**	Medium value according to the patient’s tolerance	Best tolerated	Too high value may induce glottic closure; too low value may not allow PS to be reached
**Inspiratory Trigger**	Highest sensitivity that avoid auto-trigger	Avoid ineffective effort	Auto-triggering (specially in presence of leaks)
**Cycling criteria**	Starting from 50% to lower value	Inspiratory/Expiratory ratio 1:1.5–1:2	Too short inspiratory time
**EPAP (cmH_2_O)**	4–12	Treat obstruction with active drive	Leaks; reactive glottic closure
**Respiratory Rate** **(breath/min)**	12–16	Improve gas exchanges and efficiency of sleep	Asynchronies

HNIV: home non-invasive ventilation; NMD: neuromuscular disease; PS: pressure support. EPAP: expiratory positive airway pressure.

## Data Availability

No new data were created or analyzed in this study. Data sharing is not applicable to this article.
